# International student mobility and highly skilled migration: a comparative study of Canada, the United States, and the United Kingdom

**DOI:** 10.1186/2193-1801-2-132

**Published:** 2013-03-25

**Authors:** Qianru She, Terry Wotherspoon

**Affiliations:** Department of Sociology, University of Saskatchewan, (1019 - 9 Campus Drive), Saskatoon, (S7N 5A5) Canada

## Abstract

Against the backdrop of demographic change and economic reconfiguration, recruiting international students, especially those at tertiary level, has drawn growing attention from advanced economies as part of a broad strategy to manage highly skilled migration. This comparative study focuses on three English speaking countries receiving international students: Canada, the United States, and the United Kingdom. International student policies, in particular entry and immigration regulations, and the trends in student mobility since the late 1990s are examined drawing on secondary data. By exploring the issue from the political economy perspectives, this study identifies distinct national strategies for managing student mobility, determines key factors shaping the environment of student migration in each nation, and addresses the deficiency of human capital theory in the analysis of global competition for high skills.

**Electronic supplementary material:**

The online version of this article (doi:10.1186/2193-1801-2-132) contains supplementary material, which is available to authorized users.

## Introduction

Today’s global economy is characterized as a post-industrial knowledge economy, where a nation’s “knowledge advantage” in cultivating a well-educated, highly skilled, and flexible workforce has been recognized as the most critical asset for economic prosperity (see e.g., Her Majesty’s Stationery Office HMSO 2006; Department of Finance Canada 2006). As most advanced economies are experiencing a continuous decline in working-age populations (Chaloff and Lemaître 2009), the competition for highly skilled migrant workers has become increasingly intense. From 1990 to 2000, the stock of immigrants with tertiary education (either from their home country or host country) in the member nations of the Organisation for Economic Co-operation and Development (OECD) rose by almost 8 million (Docquiera et al. 2009). The past decades also saw a tendency towards an increase in the employment of immigrants in high-skill occupations (Chaloff and Lemaître 2009). Against the backdrop of the global talent war, recruiting international students has drawn growing attention from advanced economies and has been integrated into their strategies to attract and retain highly skilled migrant workers (Organisation for Economic Co-operation and Development OECD 2008a).

Although international student mobility is emerging as a subject in research on highly skilled migration, few studies attempt to reveal distinct national strategies for managing student mobility. As human capital theory remains a key approach underlying research on skilled migration, previous comparative studies focusing on international student mobility are limited to the comparisons of governments’ performance in attracting and retaining international students without taking specific goals of recruiting international students into account (see e.g., Verbik and Lasanowski 2007). This paper offers a comparative study focusing on three principal English-speaking countries receiving international students: Canada, the United States and the United Kingdom. International student policy, in particular visa and immigration regulations, and its outcomes in terms of the trends in student mobility since the late 1990s are examined and compared drawing on secondary data. Through a discussion from the political economy perspectives, this study identifies distinct national strategies for managing student mobility, determines key factors shaping the environment of student mobility in each nation, and addresses the deficiency of human capital theory in the analysis of global competition for high skills. The paper suggests that international student migration, among other discretionary migration categories, is subject to a specific national approach to the high skill economy, which represents a collective goal of national interests, not exclusively to enhance the skill bases in the host country.

The paper begins by reviewing relevant literature on international student mobility. It then examines distinct policy frameworks for managing student mobility in each country as part of a broad strategy to manage highly skilled migration. The trends in international student mobility in the three countries are presented and compared in the following session. The Discussion highlights three distinct national strategies for managing student migration.

### International student mobility in the global knowledge economy

Several main themes can be identified with respect to the recruitment of international students as part of a broad strategy to manage highly skilled migration.

First of all, immigrant receiving nations have recognized demand-driven immigration, which targets labour market needs, as a more pragmatic way compared to a supply-driven mode of immigration, as the former is to a certain extent able to avoid imposing immediate financial burden on the receiving society (Chaloff and Lemaître 2009). Thus, there has been an increasing emphasis on an employer-oriented selection of immigration (Chaloff and Lemaître 2009). Local authorities have also begun to play a vital role in managing migration for the purpose of meeting local demand for skills (Organisation for Economic Co-operation and Development OECD 2007). With respect to admission requirements, host countries have paid more attention to newcomers’ language proficiency, work experience, and prior success which are seen as key indicators of labour market outcomes. Preference of residence approval and status change has also been given to temporary residents who have local work or study experience. As temporary residents in their host countries, international students are seen as potential skilled workers who are more easily integrated into local labour markets due to their verified credentials, country-specific experience and skills, and social connections (Tremblay 2005; Organisation for Economic Co-operation and Development OECD 2007; Chaloff and Lemaître 2009).

Secondly, previous studies have demonstrated a significant link between academic mobility and potential migration. One of the key reasons for student mobility is to acquire post-graduate employment in host countries (Suter and Jandl 2006; Rosenzweig 2008). Cross-sectional analyses reveal that many former international students undergo a shift in status from students to work permit holders or permanent residents (Suter and Jandl 2006). Approximately, 15-35% of international students can be expected eventually to work and settle in their host countries; the higher the level of education is, the more graduates stay (Suter and Jandl 2006). Meanwhile, hosting international students has a significant positive effect on future migration, regardless of previous immigration stock (Dreher and Poutvaara 2005).

Host countries’ interests in international students as a pool of highly skilled migrant workers in particular lie in the following two reasons. First, with the development of information technology and the outflow of manufacturing jobs to less developed countries, human resources in science and technology (HRST) have become a key indicator of innovation (see e.g., Organisation for Economic Co-operation and Development OECD 2008b; Organisation for Economic Co-operation and Development OECD 2009a). Compared to native-born youth, international students are more likely to be enrolled in science and technology programs where the acquired skills can be easily transferred to other circumstances (Suter and Jandl 2006). Second, international students account for a high proportion of enrolment in advanced research programs (Tremblay 2005; Suter and Jandl 2006), highlighting their potential contributions to host countries’ economies in case they stay upon completion of their study.

Thirdly, international student policy has become a tool in the global competition for high skills. OECD countries, which have been dominating in receiving worldwide mobile students, are engaged in marketing their higher education institutions, easing entry and status extension regulations, allowing international students to work during studies, and offering channels for them to change status and stay as knowledge workers (Tremblay 2005; Suter and Jandl 2006; Santiago et al. 2008; Chaloff and Lemaître 2009). The convergence in governments’ international student policy has demonstrated widely recognized benefits from educating foreigners.

In spite of the above, other facets of international student mobility need to be addressed. In the face of a rapid growth in foreign enrolment worldwide, particularly in the OECD nations, new trends in the distribution of international students have emerged. For instance, as New Zealand, France, and Japan have become more ambitious and made substantial progress in recruiting international students, the US, a traditional leading destination country, has seen a remarkable drop in its share of international higher education market (Organisation for Economic Co-operation and Development OECD 2008a). Moreover, international students are mainly from a small number of principal source regions such as China, India, and South Korea; the destinations of the students are heavily geographically oriented, with European students tending to stay in Europe and students from the rest of the world tending to study in the OECD countries outside of Europe (Organisation for Economic Co-operation and Development OECD 2007). In addition, for most host countries with data available, a very limited proportion of international students stay after the completion of their education (Suter and Jandl 2006).

The imbalanced growth and limited stay rates suggest important questions about governments’ commitment to recruiting international students. In fact, despite the convergence in international student policies across host countries, detailed regulations, procedures, and mechanisms through which policies are carried out differ from one country to another (Cornelius and Tsuda 2004). To a substantial extent, receiving countries remain in favour of their specific policy frameworks (Chaloff and Lemaître 2009), and give privilege to certain immigration categories or applicants from certain places of origin, even when it comes to the most qualified. Hence, it is critical for the discussion of international student mobility to go beyond the consideration of human capital accumulation and economic productivity and examine a broader range of political economy conditions which shape government practices of managing highly skilled migration.

### International student mobility as part of highly skilled immigration: distinct policy frameworks

Canada addressed its commitment to a high skill economy and outlined the strategies to tackle the skill challenge in its Innovation Strategy (Industry Canada 2002) ^a^ and economic plan ^b^, where the government highlighted the measures to attract the best international students through financial incentives, branding campaigns and immigration programs. Since the mid-2000s, the government has launched a series of policy initiatives to expedite the processing of study permit applications and enhance access for foreign students to Canadian labour market during and after their study. The major steps include the national roll-out for the Off-Campus Work Permit Program (Citizenship and Immigration Canada CIC 2008), the extension of the validity of Post-Graduation Work Permit to up to three years (Citizenship and Immigration Canada CIC 2009a), and the implementation of the Canadian Experience Class (CEC) (Citizenship and Immigration Canada CIC 2010a). The Provincial Nominee Programs have also been playing an increasingly important role to attract and retain international students and meet local skill needs since the late 1990s.

As a member county of the European Union (EU) and the European Economic Area (EEA), the UK has traditionally been involved in Europe-wide labour mobility and skill development programs (such as the ERASMUS programme). Besides the commitment to the European higher education market, the UK announced its strategic plans for participating as an ambitious competitor in the global higher education arena though the two Prime Minister’s Initiatives (PMI) launched in 1999 and 2006 respectively. The PMIs set specific goals of recruiting international students through marketing UK education, reducing the dependence on a small number of source countries, and ensuring the quality of student experience from application and visa process to the end of the studies (British Council 2008a). The PMI2 adopted a more strategic agenda by giving more weight to non-monetary considerations such as international influence and partnership building (British Council 2008a). In 2008, a new points-based system (PBS) was launched to regulate non-EEA nationals who apply to come to the UK to work or study. The new system streamlines the process for genuine students to study and gain work experience in the UK while guard against the risk of bogus students in order to protect the UK labour market (UK Border Agency UKBA 2008). With respect to working after studies, the evolving of student immigration categories from the Science and Engineering Graduates Scheme (SEGS) through the International Graduates Schemes (IGS) to the Post Study Work category under the Tier 1 of the PBS eased the requirements for eligible students to work in the UK after graduation (Salt 2009; Border and Immigration Agency 2007). Lately, the UK’s immigration control has reached a new high. The government has decided to scrap the Post Study Work scheme in April 2012 (Workpermit.com 2011a), and has replaced the previous Tier 1 General visa with the new Tier 1 Exceptional Talent scheme in August 2011 (Workpermit.com 2011b). These changes make it more difficult for international students from non-EEA countries to be qualified for a work permit to stay in the UK after graduation.

The US has traditionally been the most attractive destination for international students, especially for those at advanced research levels, due to its academic prestige and extraordinary education and research resources (Marginson 2006). As the US immigration shifted its preference from privileging European origins to favouring family ties and the quality of applicants as employees in the mid-1960s, the large number of foreign students and their dependents in the US were mainly seen as an economic boon for the country in both the short and long term (Martin 2004). However, the September 11 terrorist attacks triggered the tightened border security, which significantly brought down the inflows of international students due to the complex scrutiny associated with visa application (see e.g., Yale-Loehr et al. 2005; Hindrawan 2003). In order to restore the US power in bringing international students, the government undertook a number of measures since the mid-2000s to simplify and expedite visa processing. The most recent policy initiatives highlighted provisions for international students to obtain US work experience and change their status upon graduation, which include the H-1B advanced degree exemption (US Citizenship and Immigration Services USCIS 2010) and the extension of the period of the Optional Practical Training (OPT) program (Department of Homeland Security DHS 2008). However, evidence shows that even with the improvement brought in to speed the procedures after the terrorist attacks, the average visa processing time for scientists, scholars, and students has climbed up recently (see e.g., Kaplan 2009, Chiu 2009; Stone 2008).

Facing the global knowledge economy and demographic change, international student receiving countries, such as Canada, the US and the UK, have been using visa and immigration policies as a tool to attract international students and retain the best. These countries have demonstrated their openness to an ever larger number of international students by streamlining entry process, enhancing student experience, and promoting flexibility for status change. On the other hand, all three countries seek an overall control on immigration. Most explicitly, Canada’s Post-Graduation Work Permit Program, the US’s OPT program, and the UK’s Post Study Work scheme set probationary periods to test international students’ adaptation to the local labour market and to make sure that only those highly skilled who have succeeded in being integrated into the receiving society are able to eventually fulfil their intention to stay.

While international student policies in all three countries turn out to be convergent in the sense of balancing openness and control, country-specific regulations and the power that shapes the changing policy environment indicate distinct frameworks for managing student migration.

On the level of general migration policy, Canada and the US are largely seen as classic immigration countries (in comparison with “reluctant” labour importers such as the UK), in which immigration is a fundamental part of the founding myth, historical consciousness and national identity (Cornelius and Tsuda 2004). However, since the 9/11 attacks, the US has become nearly as prone as a “reluctant” labour importers to adopt restrictive measures and indulge anti-immigrant public opinion (Cornelius and Tsuda 2004). By giving policy priority to family reunification and setting quotas for most highly skilled immigration categories, the US clearly shows its general stance of quantity control over immigration (Chaloff and Lemaître 2009; Martin 2004). On the contrary, Canada’s skill-based immigration is consistent with its continuing prospect of nation-building through human capital accumulation, which allows three-times the level of per capita immigration as the US has, while at the same time maintains a stable and relatively high public tolerance to immigration (Cornelius and Tsuda 2004).

With respect to international students, the US is most selective in both bringing in and retaining international students. The country has strong preference for students in science, technology, engineering, and mathematics (SETM) areas and advanced research programs (see e.g., Department of Homeland Security DHS 2008). However, even the most qualified still face substantial difficulties in obtaining work permits or permanent residency after the completion of their study. Canada, on the other hand, offers relatively broad access to and easy process of entry and settlement (in some occasions without the need of a current job offer). The terrorist event did not provoke the same restrictive turn in Canadian immigration policy, despite its increased cooperation with the US on border security (Cornelius and Tsuda 2004). In short, regarding international student policy, the US is inclined to have low openness to international students’ entry and high control on their settlement, whereas Canada tends to act in the opposite way: high openness to entry and low control on stay.

The UK has moved towards more liberal admission of highly skilled immigrants as a consequence of an aging population and severe shortages of professional workers (Cornelius and Tsuda 2004). However, the UK is still characterized as a reluctant labour importer. The attitudes of political elites and general public towards immigration generally are more negative than in the classic immigration countries (Cornelius and Tsuda 2004). This to a large extent accounts for the facts that the country recruits most migrants temporarily as guest workers rather than as permanent additions to the labour force (Cornelius and Tsuda 2004), and it does not have a clear philosophy or proactive measures to integrate newcomers (Layton-Henry 2004).

The UK is reluctant to admit immigrants especially from non-EEA countries. Despite the observation that most entry categories of foreign nationals from outside the EEA have seen rapid increase since the mid-1990s (Layton-Henry 2004), the UK’s immigration policy, in particular skilled immigration, gives priority to nationals from the EEA community (Chaloff and Lemaître 2009). In general, the UK seeks to be less dependent on migration in the future through up-skilling domestic workers and controlling foreign inflows (Salt 2009); meanwhile, it takes advantage of the provisions of free labour movement in Europe as much as possible to meet the demand for high skills (Chaloff and Lemaître 2009).

The UK’s immigration control and preference for EEA nationals apply well to international students. Even though the country has made its first-rate higher education resources more accessible to international students from non-EEA countries, it decidedly insists on immigration control considering post-graduate status change. Combining a relatively high level openness and control in managing international student mobility, the UK’s strategy to recruit international students, in particular from non-EEA countries, is the least integrated into its skilled immigration plan compared to Canada and the US.

As was discussed above, the orientations of international student policy, in terms of entry and status change, in Canada, the US and the UK can be simplified in the terms outlined in Table 1, regardless of the actual numbers of student inflows.

**Table 1:** **Distinct policy frameworks for managing international student mobility**

		Openness to entry	
		High	Low
Control on stay	High	United Kingdom	United States
	Low	Canada	

### Trends in international student mobility

In 2008, over 3.3 million tertiary students were enrolled outside their country of citizenship (0.8 million in 1975) of whom 2.7 million (79.1%) studied in OECD nations (Organisation for Economic Co-operation and Development OECD Organisation for Economic Co-operation and Development OECD 2010a). The US, the UK and Canada ranked among the top countries receiving international students, with a combined share of 34.2% of foreign tertiary enrolment in 2008 (Organisation for Economic Co-operation and Development OECD 2010a).

### How many international students study in the three countries?

**Figure 1:**
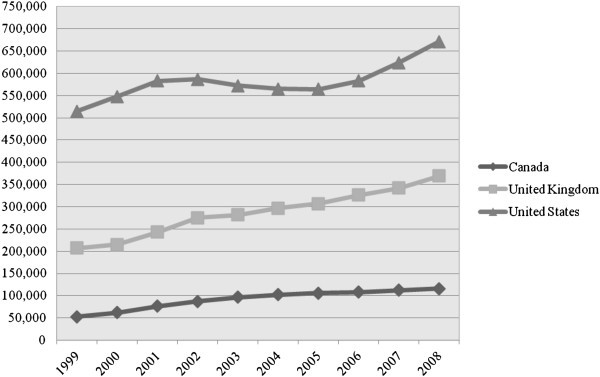
**International students in tertiary education, 1999-2008.** Data from Citizenship and Immigration Canada CIC (2010b), Higher Education Statistics Agency HESA (2010a, 2009, 2008, 2007, 2001a), and Institute of International Education IIE (2010).

**Figure 2:**
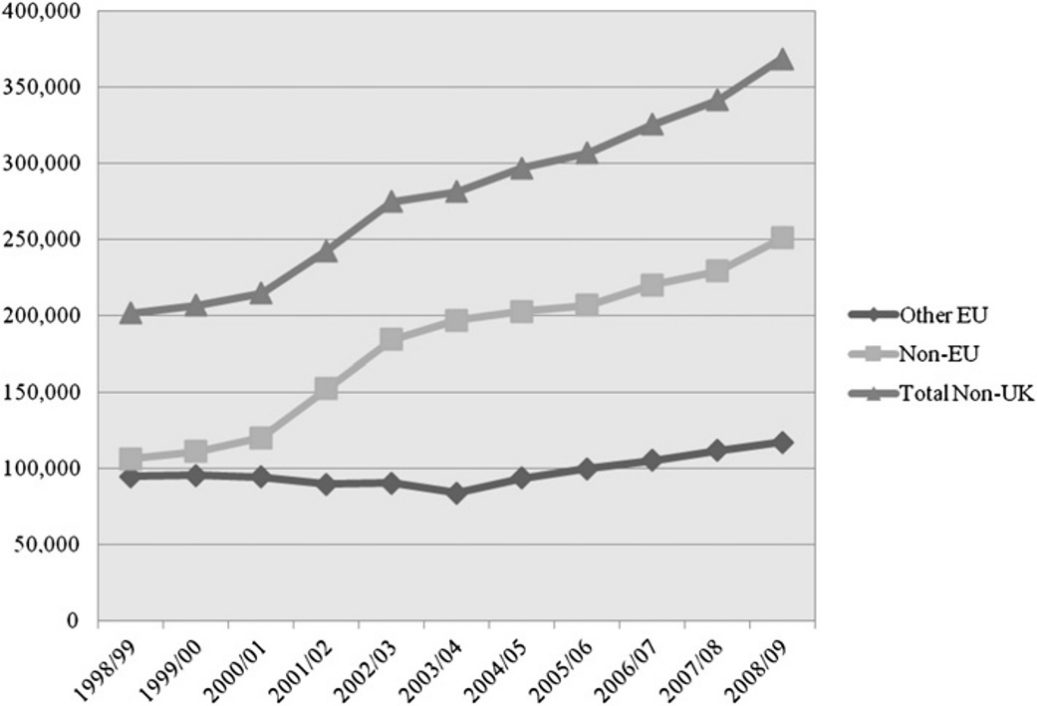
**International students by domicile, the UK, 1998/99-2008/09.** Data from Higher Education Statistics Agency HESA (2010a, 2009, 2008, 2007, 2001a).

Figure 1 shows the trends in the stock of international students in Canada, the US and the UK. The US stands highest in the number of international students and Canada the lowest. Nonetheless, Canada and the UK saw a stable growth in the stock of international students between 1999 and 2008, whereas the number of students in the US fluctuated over the period. The situation in the UK is unique in that the increase in the total number of international students relies on the growth in the number of students from non-EU countries, as shown in the split of international students by domicile (Figure 2).

**Figure 3:**
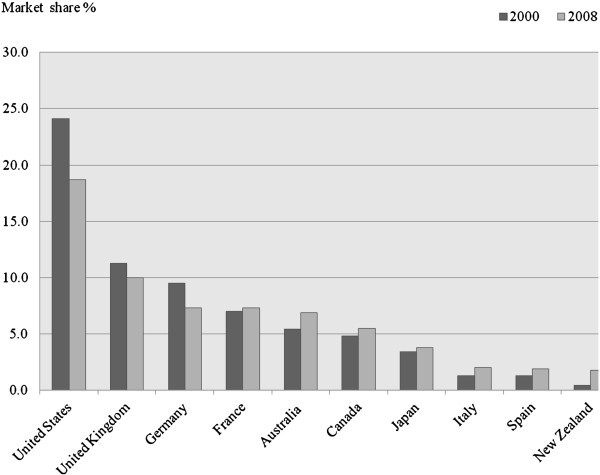
**Share of international students in selected OECD countries, 2000 and 2008.** Data from Organisation for Economic Co-operation and Development OECD (2010a); Canada data refer to the year 2007 and exclude private institutions.

Figure 3 shows market shares of the top ten OECD countries receiving international students, including Canada, the US and the UK. Clearly, the US stands on the very top among all host countries. However, the country’s share of worldwide international students declined by over 5% (from 24.1% to 18.7) between 2000 and 2008.

### How mobile are tertiary students?

**Figure 4:**
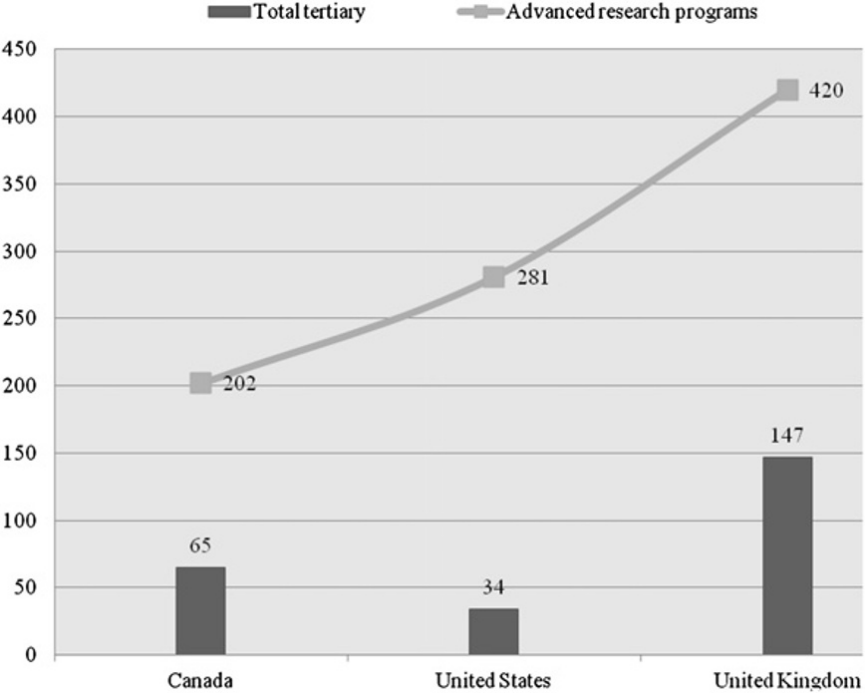
**Student mobility: International students per 1,000 students enrolled in tertiary education, 2008.** Data from Organisation for Economic Co-operation and Development OECD (2010a); Canada data refer to the year 2007 and exclude private institutions.

Student mobility can be measured by the proportion of international students in tertiary education (see e.g. Organisation for Economic Co-operation and Development OECD 2010a; Organisation for Economic Co-operation and Development OECD 2009b). Figure 4 shows that in all three countries, student mobility in advanced research programs is significantly higher than that in total tertiary education, which indicates that the three countries rely more on foreign enrolment in advanced research programs than in other levels of programs in tertiary education. The UK has the most mobile students in both total tertiary education (147 international students per 1,000 students enrolled) and advanced research programs (420 international students per 1,000 students enrolled). The US has the lowest student mobility in tertiary education, which suggests the highest domestic educational consumption compared to Canada and the UK.

### At what academic levels do international students study?

**Figure 5:**
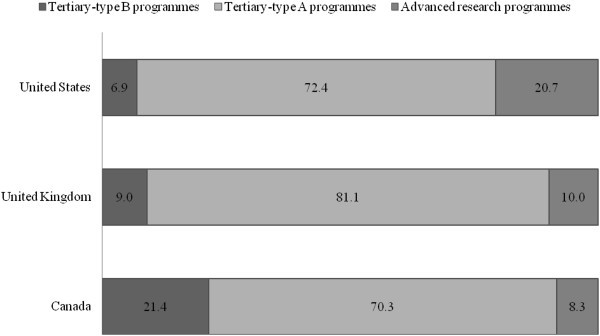
**Distribution of international students in tertiary education by academic level (percentage), 2008.** Data from Organisation for Economic Co-operation and Development OECD (2010a); Canada data refer to the year 2007 and exclude private institutions.

The OECD and the UNESCO have presented cross-country data on the types of programs attended by international students. Figure 5 displays the distribution of international students in tertiary education (type 5B, 5A and 6) in each country. It shows that international students are predominantly enrolled in programs that offer a university degree (approximately the equivalent of tertiary-type 5A and 6). Over 90% of international students in the US and the UK enrolled in tertiary-type 5A and 6 programs, whereas only 78.6% of international students in Canada studied at the same levels. Table 2 highlights the number of international students graduating from university degree programs (approximately tertiary-type 5A and 6). It confirms the US advantage in holding doctorate students and suggests that the majority of international students in Canada seek a Bachelor’s degree.

**Table 2:** **Distribution of international graduates by level of study (percentage), 2008**

	Bachelor	Master’s	Doctorate
United States	32	55	13
United Kingdom	35	59	6
Canada	61	34	5

UOE and UNESCO Institute for Statistics, cited in Kennedy (2010).

### How many students study in sciences and engineering?

Higher education in sciences and engineering (S&E) ^c^ has been widely recognized as a crucial factor of economic competitiveness. Advanced economies compete with each other in promoting their shares of total enrolment in S&E and earned S&E degrees, which are seen as indicators of national innovation performance (e.g., Organisation for Economic Co-operation and Development OECD 2009a). Table 3 presents international students enrolled in S&E at all tertiary levels and in graduate programs as percentage of total international students enrolled, calculated based on data from multiple sources. Of international students enrolled in tertiary education in Canada, the US and the UK, a considerably large proportion (compared to the proportion among domestic students) enrolled in S&E with the lowest in Canada at 32.3% and the highest in the UK at almost 38%; the percentage of international students at graduate levels of study was even higher. Almost half (49.7%) of international graduate students in the US enrolled in S&E in 2006, which can be expected to yield the highest number of foreign graduate students enrolled in S&E (see also Table 4 below) given the US share of worldwide mobile students and the country’s strength in attracting advanced researchers.

**Table 3:** **Foreign students enrolled in S&E, 2006**

	Total foreign S&E students	Total foreign students	Foreign S&E/Total foreign (%)^a^	Foreign S&E Graduate	Foreign graduate	Foreign S&E graduate/Total foreign graduate (%)^a^
Canada	34,270	106,058	32.3	13,060	-	-
UK	123,580	325,985	37.9	67,630	158,920	42.6
US	201,765	582,984	34.6	131,455	264,288	49.7

National Science Foundation NSF (2010, Table 2-45; 2008, Table 2-45, 2-24); Citizenship and Immigration Canada CIC (2010b); Higher Education Statistics Agency HESA (2010a); Institute of International Education IIE (2010); Canada data refer to the year 2005.

^a^ Calculated by authors.

**Table 4:** **Foreign graduate students enrolled in S&E, 1995 and 2006**

	Foreign S&E graduate students		Foreign S&E graduate students/ Total S&E graduate students (%)		Total% change^a^	Annual% change^a^
	1995	2006	1995	2006	1995 to 2006	
Canada	7,690	13,060	17.0	19.8	69.8	7.0
UK	28,850	72,360	28.9	45.1	150.8	13.7
US	102,885	151,018	20.6	25.3	46.8	4.3

National Science Foundation NSF (2010, Table 2-45, 2-43, 2-17; 2008, Table 2-45); Canada data refer to 1995 and 2005.

^a^ Calculated by authors.

Table 4 displays the growing mobility of graduate students enrolled in S&E in each country according to data from US National Science Foundation (NSF). All three countries experienced a growing reliance on foreign graduate enrolment in S&E. The UK had a 13.7% annual growth in the number of foreign graduate students in S&E, exceeding both Canada (7.0%) and the US (4.3%). Meanwhile, the UK had the highest percentage of foreign graduate students in S&E out of all graduates students enrolled, as well as the fastest growth in the percentage (from 28.9% to 45.1%). In other words, the UK has the most mobile graduate students in S&E and the most rapid growth in the mobility compared to Canada and the US.

### Where Are international students from?

Table 5 presents the top ten countries/regions sending international students to each of the three countries in 2008. Clearly, students from Asia accounted for the largest proportion of international students in all three countries. Meanwhile, the places of origin of international students featured sending countries that are close to the receiving ones historically (such as former colonies to the UK), geographically (e.g., the US to Canada and vice versa), economically (e.g., other EU countries to the UK) or/and culturally (e.g., France to Canada).

**Table 5:** **Top 10 counties/regions sending international students to Canada, the US, and the UK, 2008**

	Canada		United States		United Kingdom	
	Country of origin	% as total international students	Country of origin	% as total international students	Country of origin	% as total international students
1	China	23.7	India	15.4	China	13.8
2	South Korea	15.4	China	14.6	India	10.0
3	United States	6.4	South Korea	11.2	Ireland	4.5
4	France	4.8	Canada	4.4	Nigeria	4.2
5	India	4.1	Japan	4.4	United States	4.2
6	Japan	3.7	Taiwan	4.2	Germany	4.1
7	Saudi Arabia	2.6	Mexico	2.2	France	3.8
8	Taiwan	2.3	Turkey	2.0	Malaysia	3.7
9	Hong Kong	2.3	Vietnam	1.9	Greece	3.5
10	Mexico	2.2	Saudi Arabia	1.9	Pakistan	2.8
	Total top 5 2008^a^	54.3	Total top 5 2008^a^	50.0	Total top 5 2008^a^	36.6
	Total top 5 1999^a^	46.2	Total top 5 1999^a^	41.6	Total top 5 1999^a^	39.4

Citizenship and Immigration Canada CIC (2009b); Higher Education Statistics Agency HESA (2010b, 2001b); Institute of International Education IIE (2010); Canada data include foreign students in educational programs at all levels.

^a^ Calculated by authors; The top five sending countries and their ranks may change over years.

However, the extent to which host countries rely on their leading source countries varies. For instance, the proportion of international students from the top five source countries increased in Canada (from 46.2% to 54.3%) and the US (from 41.6% to 50.0%), but decreased in the UK (from 39.4% to 36.6%) during the past decade (Table 4). The figures suggest a reduced reliance of the UK on traditional source countries for foreign enrolment compared to Canada and the US. It should be noted that the emerging source countries of international students in the UK (such as China, India, and Nigeria) highlight the nation’s success in marketing its higher education in priority countries exclusively in non-EEA areas (see British Council 2008b), which corresponds to its shifting attention from emphasizing numbers to a broader strategy of global partnership and influence.

### How many international students stay after graduation?

International evidence of student retention can largely be drawn from two sets of data: the first one is retention rate, which is roughly the proportion of international students who gain residence in the country, ideally by cohort; the second is the proportion of residence approvals who are former students in the host country (Merwood 2007). Very few countries are able to provide such evidence; meanwhile, existing data need to be used and compared with caution due to country-specific methods of measurements and data collections.

Approximately 15%-20% of international students eventually settle and work in Canada, and the large majority of the students changed their status for work-related reasons (see Suter and Jandl 2006; Organisation for Economic Co-operation and Development OECD 2010b). The proportion of transition made by international students to either temporary or permanent residents has increased significantly. For instance, in 2009, 8.9% of the transition to foreign workers was made by foreign students versus 2.2% in 2000. ^d^ Preliminary data show that, in 2009, 74% of all admissions through the CEC were former students (Word Education Services WES 2010), and the approval rate under the student stream was 86% in 2010 (Citizenship and Immigration Canada CIC 2010c). Additionally, the number of international students, spouses and dependents admitted through Provincial Nominee Programs grew by 128% between 2005 and 2008 (Word Education Services WES 2010).

The UK has very limited data regarding temporary status change of international students from non-EEA countries. In 2007, 2,243 students were approved for the SEGS which was only 0.5% of the total foreign labour immigration (Salt 2008). In 2008, 16,171 international students entered the UK labour market through the IGS, comprising 4.2% of the country’s total labour immigration (Salt 2009). The number went up to 34, 958 in 2009 accounting for 8.6% of the total labour immigration as the Post Study Work category was phased in, but dropped off to 12,637 in 2010 (Salt 2010), which might be associated with the recently rising immigration control.

The US does not have firm data on retention rates of international students except for foreign S&E doctorate recipients (Organisation for Economic Co-operation and Development OECD 2009c). Finn (2010) reported that the ten years stay rate of foreign doctorate recipients was 60% in 2007, which had not declined but reached a new high compared to the previous six-year period. The US Immigration and Naturalization Service USINS (2000) reported that 23% of the approved H-1B petitions were for those who had a prior student visa; of all those who transferred from a non-immigrant status to H-1B holders, approximately 58% were F-1 students in the country.

## Discussion

International comparisons have drawn a comprehensive picture of international student migration in Canada, the US and the UK. On the one hand, the magnitudes of student mobility in all three countries have increased significantly over the past decade. The increase has been especially remarkable in the number of students enrolled in S&E and advanced research programs. Asian countries, in particular the most populated ones, have enriched the flourishing academic movement by dominating the lists of the top sending nations for all the three countries.

On the other hand, there are evident gaps among governments’ performance in attracting and retaining international students. Following the 9/11 attacks, the US experienced the first consecutive annual drop-off over the past half a century (Institute of International Education IIE 2010). Along with a shrinking share of worldwide mobile students, the US also saw a growth rate of foreign graduate students enrolled in S&E lagging behind the UK and Canada, though it surpassed the two in the number of the students enrolled. Yet, holding nearly one-fifth of the word mobile students in its higher education, the US has never lost its first place in the competition, and it remains the first choice for advanced researchers and scientists.

The UK is outstanding in almost all aspects of recruiting international students regardless of student retention. Noticeably, the country has seen rapid growth in the number of students from non-EU countries, which now constitute two thirds of its stock of overseas students. However, all the evidence on students’ status change points to an emphasis on temporary rather than permanent change. The nation’s keenness on holding international students, in particular those from non-EU countries, no longer persists upon the completion of their studies.

With the fastest growth in foreign enrolment over the past decade among the three, Canada has demonstrated its potential in attracting international students. Although the country holds only 5.5% of total international students in the world, it has almost twice the number of the students the US has per 1,000 students enrolled in tertiary education. Canada’s advantage in managing international student mobility is most prominent in post-graduate retention, as employment-related policy provisions and new channels to permanent residency have facilitated students’ long-term stay. Still, it is disadvantaged compared to the US and the UK in the stock of international students and the proportion of international students in advanced research programs. The expansion of foreign enrolment in Canadian higher education also relies heavily on a few major source countries in Asia.

While a strategic consideration of securing the supply of highly skilled labour is generally embodied in all three countries’ practices of recruiting international students, huge variations in international student policy and its outcomes make explicit distinct national strategies to manage student migration and suggest specific goals each country seeks to achieve.

### Recruiting international students: an emerging strategy for Canada’s skill-based immigration

Canada is one of the very few countries offering international students direct paths to permanent residency, which demonstrates its far-going concern of securing future intakes of high-qualified landing immigrants (Bond et al. 2007). As part of its strategy to cope with the skill challenge, Canada’s international student policy is consistent with its emphasis on a skill-based immigration. Given the current shift of the selection of economic immigrants from the federal government to provincial administrations through the Provincial Nominee Programs, international students, especially those with a Master’s or PhD degree, can be expected to be more valued and integrated into Canada’s innovation strategies. Though there has been a growing tendency towards a demand-driven immigration selection regarding post-study retention signalled by the requirements of Canadian work experience or a current job offer, the labour market orientation in the selecting criteria in fact serves the national goal of skill upgrading and human capital accumulation due to the employment-related policy provisions.

Yet, Canada’s openness to international students and the strategic consideration of retaining the best have only recently been placed on its policy agenda for a high skill economy. As a traditional immigration country, Canada’s skill-based immigration places strong emphasis on direct import of talent from abroad, rather than on those who transfer from existing temporary categories, such as international students. ^e^ Moreover, without a fully articulated nation-wide strategy in place, recruiting international students has been conventionally managed by academic institutions themselves as a way of obtaining additional revenue and enhancing the overall offer of higher education to local residents (see Keeley 2009; Le-Ba 2007). Therefore, the recent hike in its foreign enrolment and post-graduate status change is rather prominent in the history. It can be seen as an unintended effect of the 9/11 attacks and indicates a new approach in the country’s skill upgrading. In the long run, the traditional goal of educating worldwide mobile students, especially revenue generation, will remains a fundamental one, while the increasingly rapid growth in the transitions from international students to permanent residents either directly or through pathways is emerging as a component of Canada’s skill-based immigration.

### UK: differentiated strategies and regimes of managing international student mobility

With proactive and well-developed recruiting strategies and the British government being the main actor promoting the recruitment of international students, the UK has become a big gainer in the global market of higher education. Yet, student mobility in the UK is distinct from Canada and the US in that overseas students in the UK is a mixture of those from within and outside of the European free movement area, and the practices of recruiting overseas students from the two source regions follow completely different migration regimes. Student migration among European nations largely falls in the indiscretionary category, which is part of the Europe-wide labour mobility and skill upgrading strategies managed at the European level. Whereas, student migration from outside of Europe is subject to national governance, and it is in fact the focus of almost all the most publicized migration policy adjustments and transformations recently initiated by the British government.

The UK has opened doors widely to non-EEA students, but remains reluctant to advance labour immigration from countries outside of Europe. In particular, although the opportunities for non-EEA students to gain UK work experience have been integrated into the overall offer of UK higher education, they are intended to attract overseas students pursuing enhanced experience of study abroad, rather than to facilitate post-graduate retention and labour movement. While the practice of recruiting international students from European countries is in line with the logic of developing a high skill economy, the UK’s approach to non-EEA students is mainly based on traditional consideration on the benefits of educational internationalization.

Not surprisingly, the UK intends to take more effective measures through the new PBS to facilitate the entry of genuine students who have a clear intention to leave upon graduation while guarding against the risk of bogus students who do not fit the goal. The policy provisions put in place by the British government to ease the entry of overseas students from non-EEA countries are indeed preconditioned by temporary residency and a non-settlement regime with a clear goal of protecting UK and European labour markets. Thus, the open doors to non-EEA students, stricter control on their post-graduate status change, and the priority given to EEA nationals as a pool of skilled labour coexist as three interrelated facets of the UK’s strategy to manage international student mobility.

### United States: employment-based retention and reactive practices of managing international student mobility

The US experience of recruiting international students is unique in its reactive practices of managing student mobility. In response to the rising concern about border security, the traditional open door policy was ended by the implementation of tightened visa requirements for international students and exchange visitors. Facing widespread criticisms about the negative impact of its international student policy on the US economy, especially the lobbying efforts by academic institutions and employer associations against the loss of talent, the government revisited its narrow mindset on national security and took big steps to reform visa and immigration policies in order to restore the US power in bringing international students. The US position in the global market of higher education has not changed vitally as it is still the leading destination for worldwide students, especially for those in S&E and advanced research programs. However, with no proactive measures in place, the US dominance has been challenged by its determined counterparts, such as Canada and the UK. The lack of forward-looking ability makes the US immigration policy a main obstacle to the first mover advantage in the global talent war.

Besides the US advantage in domestic and international higher education, the key factor behind its reactive recruiting policy and practices is the employment-based post-study retention. In the US, non-immigration categories (temporary residents) is an important pool of approved status change to either other non-immigrant categories or permanent immigrants. Up to 90% of foreigners receiving immigrant visas for employment are already in the US, especially via the pathway from students in higher education (OPT program), through H-1B visa holders, to permanent residents (Dunnett 2010). This pattern indicates that the decline in the inflow and stock of international students to a considerable extent leads to the reduction in the supply of high-qualified graduates for the knowledge sectors. Not surprisingly, the US corporations are among the main advocates for a liberal immigration stance to attract foreign talent including international students.

The US reactive approach to international student migration has also been demonstrated through the absence of proactive measures to adjust its retention policies as a way to attract foreign talent, which is also closely related to the employment-based immigration selection. Though the difficulties associated with the issue of H-1B visas and settlement in the US have been widely criticized, the tight control on residence approvals is not a major obstacle for the country to maintain its attractiveness to international students. For those highly qualified graduates who decidedly want to stay in the country, the status change may not be the most critical concern as the pathway to settlement is embodied in the US employment-based immigrant selection in spite of the long probationary time being non-immigrant residents. The nation has faced little trouble in achieving its immigration goals while avoiding hostile public opinion towards status change of international students, as a significant portion of this temporary category go through an employment-based selection. ^g^ Therefore, the US did not confront the urgency of reforming its student retention policy; the recent policy provisions associated with post-study work and status change, such as the H-1B advanced degree exemption and the extension of the period of the OPT program, were not initiated as proactive measures to attract international students, but rather additional offers besides the relaxed visa policy in order to re-establish the US popularity and its welcoming image to international students.

This research suggests that rather than strictly following a single logic of global human capital, nation states address and cope with the skill challenges in a strategic and political way. The management of international student mobility, among other national strategies aiming at the high skill economy, never exists simply as an economic issue of productivity, but embraces a collective goal of national interests. Host countries’ governments in fact seek to balance the concerns of different stakeholders in order to sustain and strengthen the government’s authority and legitimacy. Therefore, it is not so much whether one immigration system is more effective than another in bringing more skilled workers, but how a country can ensure that its selection criteria are consistent with its specific objectives (Organisation for Economic Co-operation and Development OECD 2007). Managing international student mobility as part of the strategy to manage highly skilled migration goes beyond merely a matter of skill formation and in fact represents specific social relations and power struggles in each host nation.

## Endnotes

^a^In 2002, Canada’s Ministry of Industry and Human Resources Development released Canada’s Innovation Strategy in two papers: *Achieving Excellence: Investing in People, Knowledge and Opportunity* and *Knowledge Matters: Skills and Learning for Canadians.* Accessible from http://www.publications.gc.ca.

^b^In 2006, Canada’s government developed a long-term economic plan *Advantage Canada: Building a Strong Economy for Canadians*. Accessible at http://www.fin.gc.ca/ec2006/plan/pltoc-eng.asp.

^c^According to the US National Science Foundation (2010), S&E includes Physical/biological sciences, Mathematics/computer sciences, Agricultural sciences, Social/behavioral sciences, and Engineering. It is to some extent different from the Organisation for Economic Co-operation and Development OECD (2009a, p. 132) definition, in which Science degrees include life sciences, physical sciences, mathematics and statistics, and computing; Engineering degrees comprise engineering and engineering trades, manufacturing and processing, and architecture and building. The OECD definition does not include programs such as agricultural and social/behavioral sciences as are contained in the NSF definition.

^d^Calculated by authors based on data presented by Citizenship and Immigration Canada CIC (2010b).

^e^Recently, Canada started paying more attention to the image of its higher education and branding the quality and values of its education for international students at varied levels of study. This may indicate future change in its national marketing strategy (see http://imagine.cmec.ca/en/understanding/).

^f^The consequent soaring inflows of international students to Canada after the terrorist attacks drew attention to a broad range of potential benefits from educating foreigners, especially the strategic role international students play as a pool of skilled workers.

^g^Though there are indeed negative evaluations of foreign student programs in the US (see Borjas 2002).

## Authors’ original submitted files for images

Below are the links to the authors’ original submitted files for images. Authors’ original file for figure 1 Authors’ original file for figure 2 Authors’ original file for figure 3 Authors’ original file for figure 4 Authors’ original file for figure 5 Authors’ original file for figure 6
