# Supplementary figures and images for: International student mobility and highly skilled migration: a comparative study of Canada, the United States, and the United Kingdom
Source: Springerplus. 2013 Mar 25;2(1):132. doi: 10.1186/2193-1801-2-132 (PMC3648681; doi:10.1186/2193-1801-2-132)

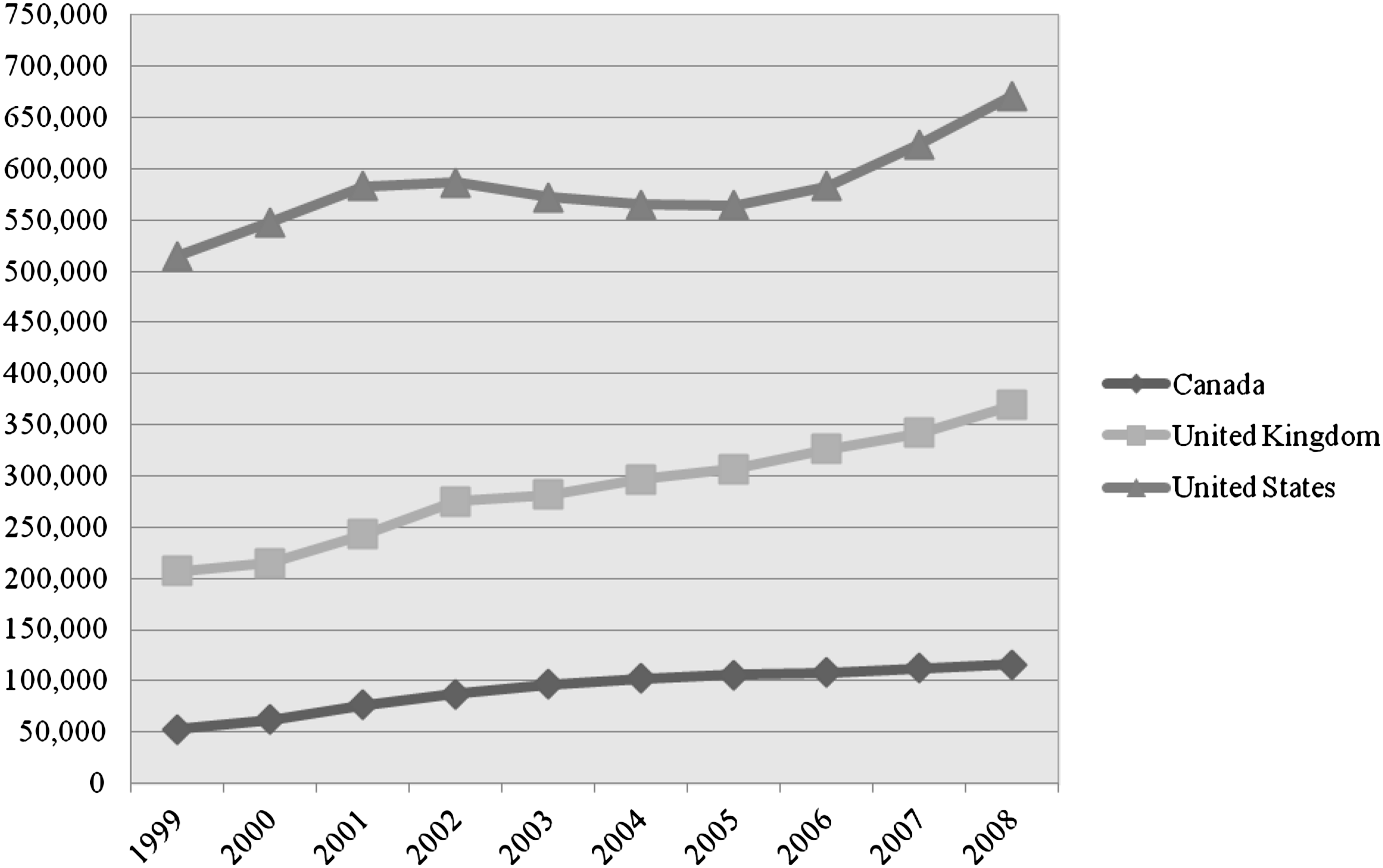

Supplement: Supplementary file 1 — Authors’ original file for figure 1 [file 40064_2013_245_MOESM1_ESM.tiff]

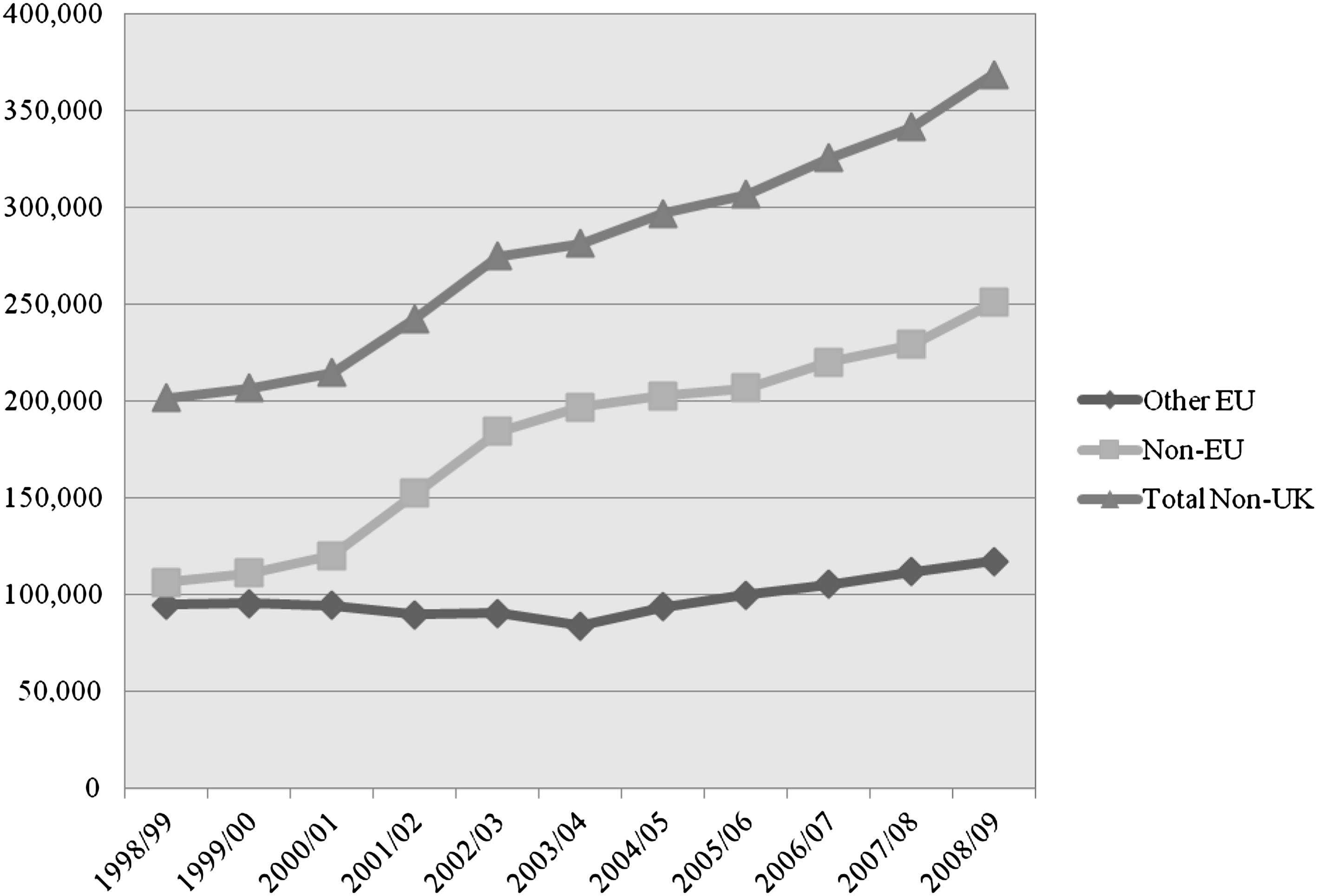

Supplement: Supplementary file 2 — Authors’ original file for figure 2 [file 40064_2013_245_MOESM2_ESM.tiff]

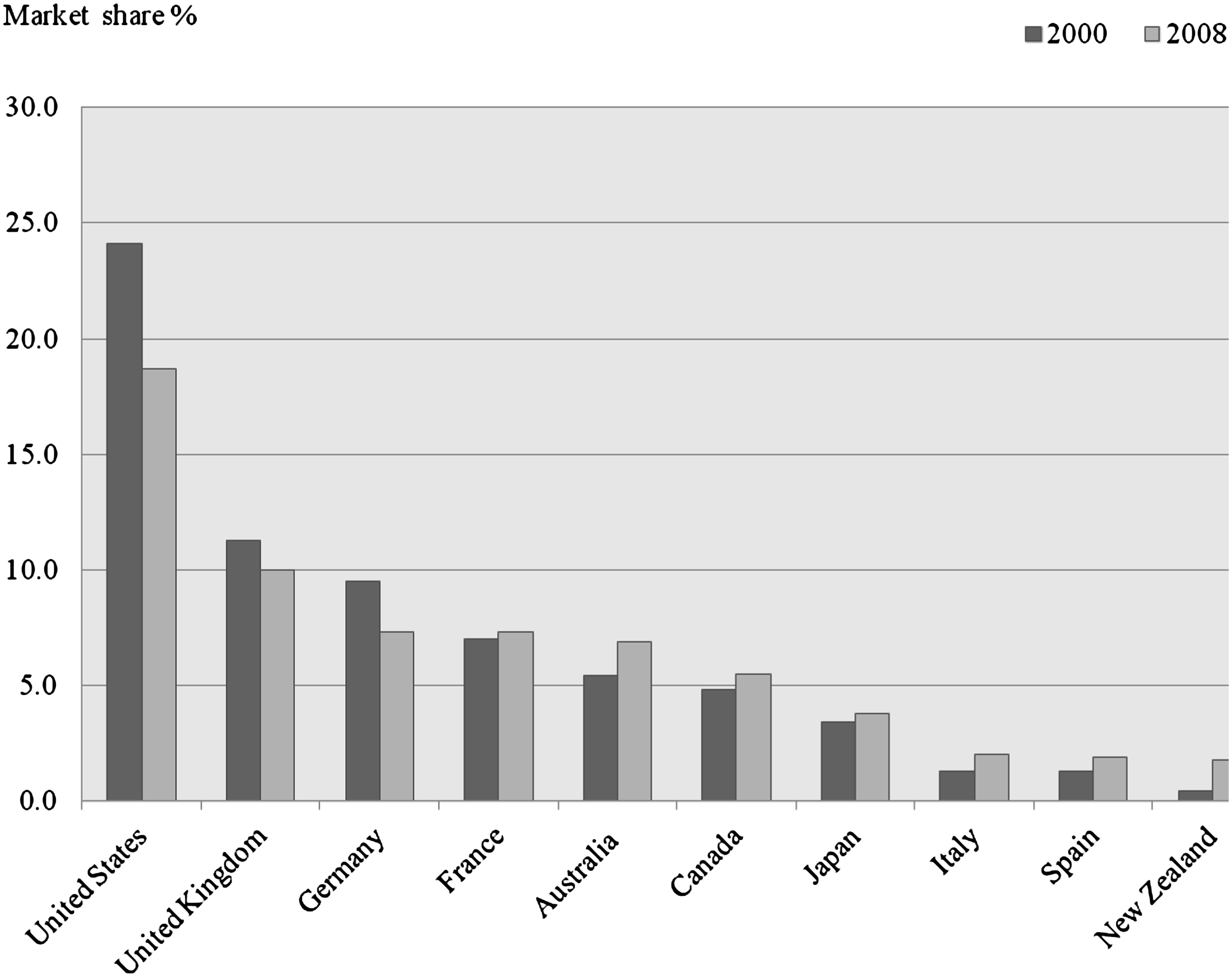

Supplement: Supplementary file 3 — Authors’ original file for figure 3 [file 40064_2013_245_MOESM3_ESM.tiff]

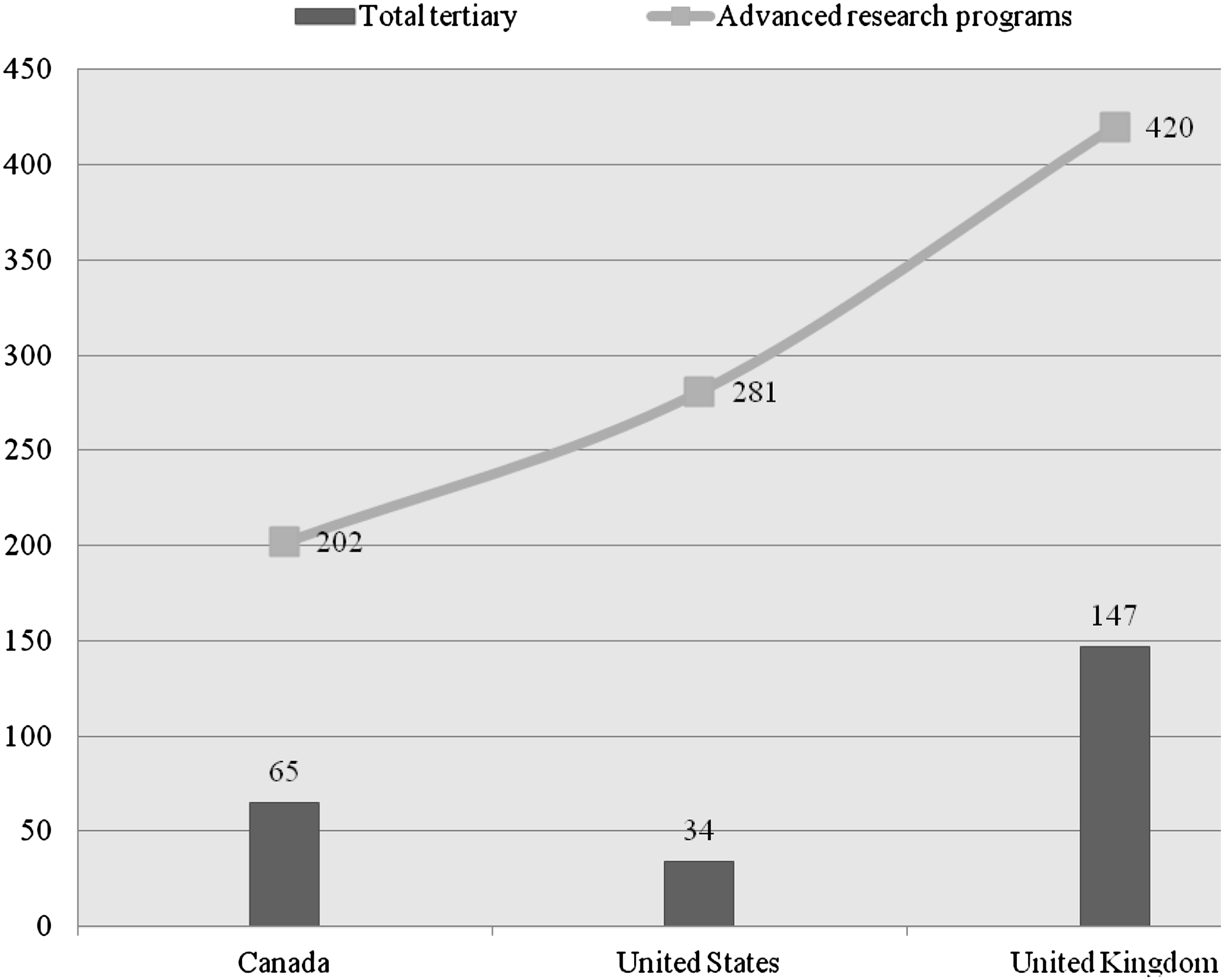

Supplement: Supplementary file 4 — Authors’ original file for figure 4 [file 40064_2013_245_MOESM4_ESM.tiff]

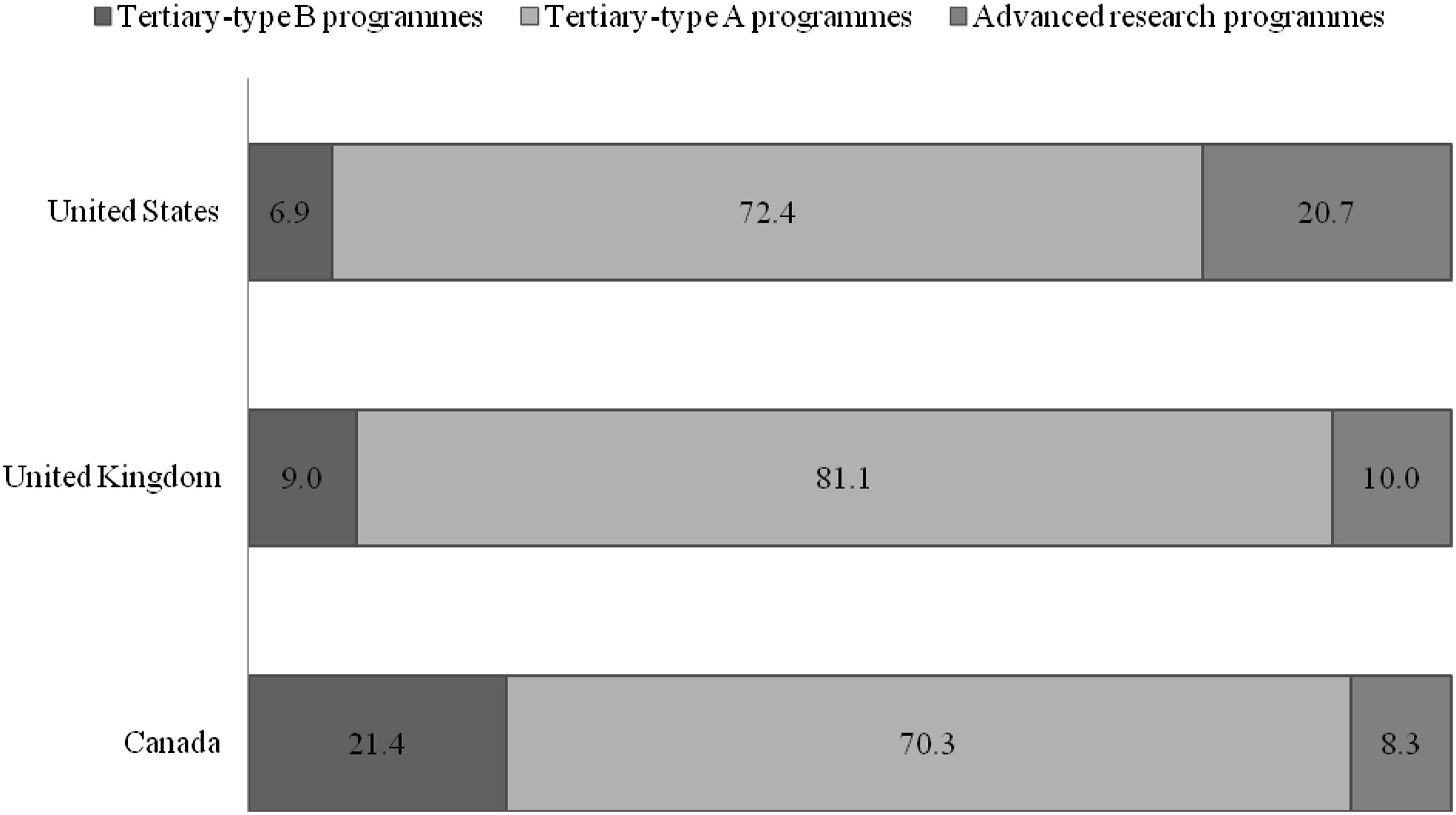

Supplement: Supplementary file 5 — Authors’ original file for figure 5 [file 40064_2013_245_MOESM5_ESM.tiff]

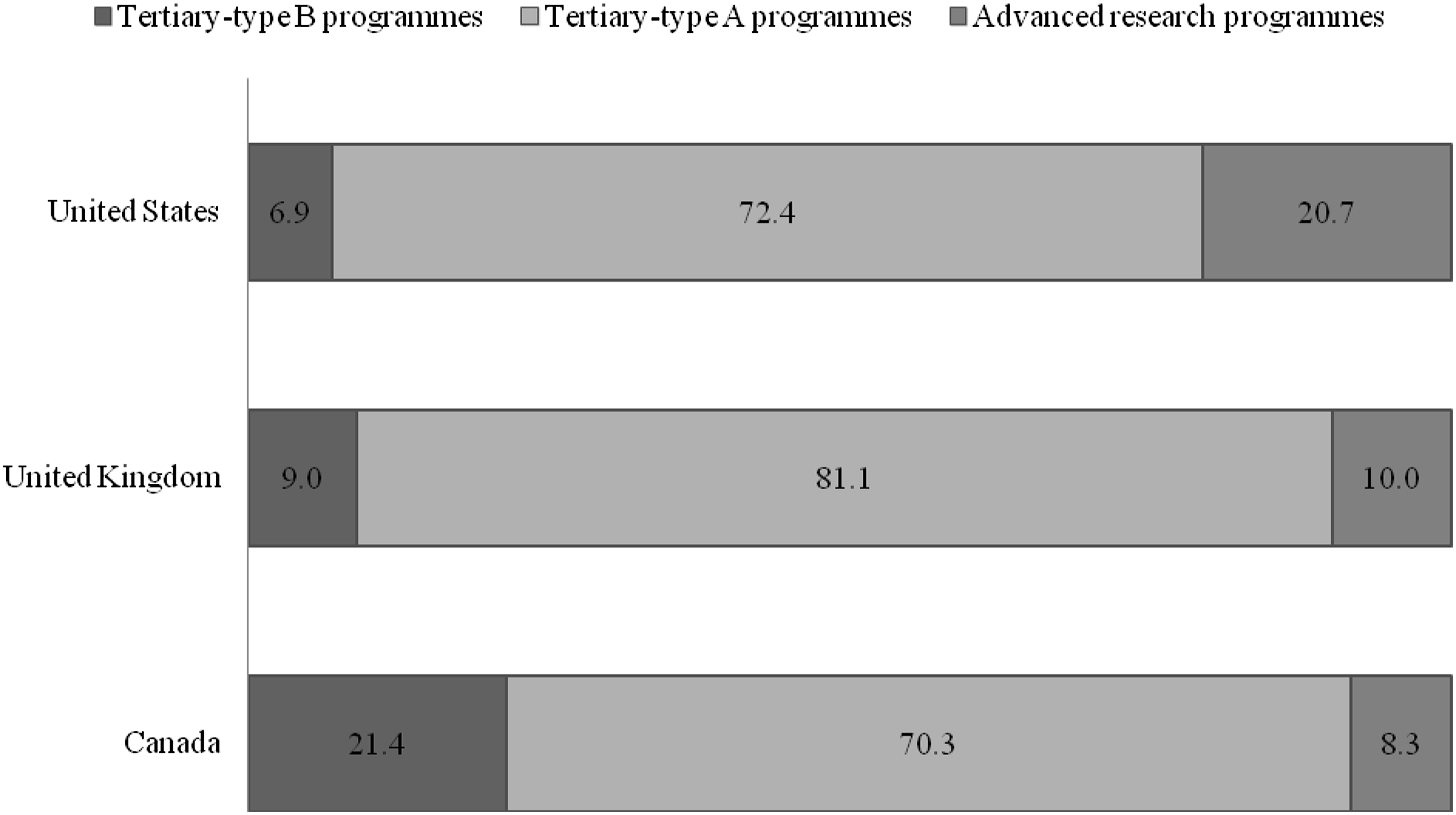

Supplement: Supplementary file 6 — Authors’ original file for figure 6 [file 40064_2013_245_MOESM6_ESM.tiff]
